# Gender Differences in Levodopa Pharmacokinetics in Levodopa-Naïve Patients With Parkinson’s Disease

**DOI:** 10.3389/fmed.2022.909936

**Published:** 2022-05-31

**Authors:** Valeria Conti, Viviana Izzo, Maria Claudia Russillo, Marina Picillo, Marianna Amboni, Cesa L. M. Scaglione, Alessandra Nicoletti, Ilaria Cani, Calogero E. Cicero, Emanuela De Bellis, Bruno Charlier, Valentina Giudice, Gerardina Somma, Graziamaria Corbi, Paolo Barone, Amelia Filippelli, Maria Teresa Pellecchia

**Affiliations:** ^1^Clinical Pharmacology Unit, Department of Medicine, Surgery and Dentistry “Scuola Medica Salernitana”, University of Salerno, Baronissi, Italy; ^2^Neuroscience Section, Department of Medicine, Surgery and Dentistry “Scuola Medica Salernitana”, University of Salerno, Baronissi, Italy; ^3^IRCCS “Istituto delle Scienze Neurologiche di Bologna”, UOC Neurological Clinic, Bellaria Hospital, Bologna, Italy; ^4^Neurologic Unit, AOU “Policlinico-San Marco”, Department of Medical, Surgical Sciences and Advanced Technologies, GF Ingrassia, University of Catania, Catania, Italy; ^5^Department of Medicine and Health Sciences, University of Molise, Campobasso, Italy

**Keywords:** gender, Parkinson’s disease, levodopa, pharmacokinetics, motor/non-motor fluctuations, dyskinesia, body weight, body mass index

## Abstract

**Background:**

Levodopa (LD) is the most effective drug in the treatment of Parkinson’s disease (PD). Unfortunately, prolonged use of LD leads to complications, mainly motor/non-motor fluctuations (MNMF) and dyskinesias (DYS). Women seem more prone to develop such LD-related complications. Nonetheless, there is a paucity of prospective studies examining gender-related predictors of MNMF and DYS. Among several factors, which concur with a very complex scenario, changes in LD pharmacokinetics influence the drug’s effectiveness. The present study aimed to assess gender-related differences in LD pharmacokinetics in patients with PD at their first-ever intake of LD.

**Materials and Methods:**

This is a multicentric study enrolling patients with PD, who were LD-naïve and received a single dose of LD/benserazide (100/25 mg) formulation. All participants gave their written informed consent, and the study was approved by the local Ethics Committees. To measure plasma LD concentrations and pharmacokinetic parameters (AUC, Cmax, Tmax, *t*_1/2_), fasting blood samples were collected before drug intake and then at 8-time points until 260 min. LD concentrations were measured by ultra-high-performance liquid chromatography coupled with mass spectrometry (UHPLC-MS). Multiple linear regression analyses were performed to identify the predictors of the parameters.

**Results:**

Thirty-five patients (16 women and 19 men) were consecutively enrolled. Area under curve (AUC) and maximum plasma concentration (Cmax) were significantly higher in women than men (*p* = 0.0006 and *p* = 0.0014, respectively). No statistically significant difference was found regarding Tmax and *t*_1/2_. Multiple linear regression analyses revealed that female sex (β = 1.559116, 95% CI 0.8314479 2.286785; *p* < 0.0001) and body mass index (BMI) (β = −0.0970631, 95% CI −0.1733004 −0.0208258; *p* = 0.014) significantly predicted AUC. Only female sex significantly predicted Cmax (β = 1,582.499, 95% CI 731.581 2,433.417; *p* = 0.001). Moreover, only BMI significantly predicted *t*_1/2_ (β = 0.0756267, 95% CI 0.0143407 0.1369126; *p* = 0.017). Stratifying by gender, BMI was confirmed to significantly predict *t*_1/2_ in women (β = 0.1300486, 95% CI 0.0172322 0.242865; *p* = 0.027), but not in men.

**Conclusion:**

This study provides novel insights on gender differences in LD pharmacokinetics, possibly contributing to the later development of motor complications and dyskinesia in PD.

## Introduction

Levodopa (LD), combined with dopa-decarboxylase inhibitors (DDCI) carbidopa or benserazide, remains the gold standard of therapy in Parkinson’s disease (PD) since the 1970s (1).

In the early stages of the disease, LD/DDCI formulations are well tolerated and are so effective in controlling the main PD-associated symptoms with a favorable benefit-to-risk ratio that the expression “honeymoon period” is commonly used (2).

Unfortunately, prolonged use of LD leads to complications, mainly motor/non-motor fluctuations (MNMF) and dyskinesia (DYS), which are among the most important determinants of patients’ disability (3).

Since plasma LD concentrations are strongly linked with drug effects, it is important to better investigate the LD pharmacokinetics (PK) to improve the drug efficacy and safety (4). In particular, increased LD absorption during chronic administration may contribute to the wearing-off phenomenon (5). Moreover, the risk for developing DYS is related to the patients’ drug exposure and is reported to rise mostly at LD dosages greater than 4 mg/kg (6).

Moreover, the occurrence of LD-related complications is dependent on several other factors, including age at onset, disease duration and severity, the length of treatment, body weight (BW), and not the least, gender. The latter is one of the most important factors since women are more prone to develop LD-related complications compared with men (7). Women show higher LD bioavailability than men as assessed by higher values of the AUC and maximum plasma concentration (Cmax) (8, 9).

Nonetheless, there is a dearth of prospective studies examining the relationship between adverse events and LD PK. Moreover, no previous study has investigated factors possibly influencing plasma LD concentrations and PK parameters in female and male patients with PD assuming LD for the first time.

In this paper, we aim to present baseline gender differences in plasma LD concentrations and PK parameters in LD-naïve patients with PD enrolled in a 2-year multicentric prospective study, which was designed to assess predictors of the development of NMMF and DYS according to gender.

## Materials and Methods

The present investigation is part of a 2-year Italian multicentric study aimed to investigate gender-related predictors of the development of MNMF and DYS in patients who are LD-naïve. The study was approved by the Ethics Committees of the participating centers (n.4_r.p.s.o./2019 for the Coordinating Center of Salerno). All participants gave their informed consent.

Thirty-five patients with PD, diagnosed using MDS clinical diagnostic criteria (10), were consecutively enrolled at the Center for Neurodegenerative Diseases (CEMAND), Department of Medicine, Surgery and Dentistry “Scuola Medica Salernitana,” University of Salerno-Italy; Movement Disorders Centre, Hermitage-Capodimonte, Naples; Dipartimento “G.F. Ingrassia,” Neuroscience Unit- University of Catania-Italy; I.R.C.C.S.- “Istituto di Scienze Neurologiche and DIBINEM”- “Alma Mater Studiorum”–University of Bologna-Italy. The questionnaire for eating habits “Grana Padano nutritional observatory” was administered (11).

All patients were LD-naïve and received a single dose of LD/benserazide (100/25 mg) formulation.

### Pharmacokinetics of Levodopa

Pharmacokinetic analysis was centralized at the Clinical Pharmacology Unit, University Hospital of Salerno.

Samples of venous blood were collected in EDTA-2Na, in fasting condition, through an indwelling catheter before and 20, 40, 60, 80, 125, 170, 215, and 260 min after drug intake.

Plasma was obtained by centrifugation (3,000 *g* for 10 min) and stored in new EDTA-2Na vacutainers at −80°C until further analysis.

The LD concentrations were measured by UHPLC-MS after protein precipitation of plasma samples, using a mixture composed of 10% TFA/1% HFBA. An appropriate volume of internal standard (ISTD) was added to the precipitation mixture at a final concentration of 2.5 μg/ml. LD was extracted from 50 μL of plasma and added to 150 μL of precipitation mixture containing ISTD. After first centrifugation (16,000 × *g*, 10 min, 4°C; 5415R Sigma-Aldrich), 150 μL of supernatants were recovered and centrifuged for other 5 min at the same speed, and 100 μL of clear supernatants were transferred to clean glass vials. The analysis was carried out on a Thermo Scientific TSQ Endura triple quadrupole mass spectrometer coupled to a Dionex UltiMate 3000 UHPLC system (Thermo Fisher Scientific, Milan, Italy) equipped with a Kinetex PFP column (50 × 2.1 mm: 2.6 μm particle size) (Phenomenex, Torrance, CA, United States). LD elution was obtained by using a two-component mobile phase, consisting of a solution (A) of 0.1% formic acid in water and (B) of 0.1% formic acid in acetonitrile used in a gradient mode (from 0 to 30% of B in 2 min). Then, the column was washed for 1 min at 70% of B and restored to the initial condition for column equilibration. The total runtime was 4.5 min. The flow rate was 0.4 ml/min and the column temperature was set at +20°C. The injection volume was 5 μL and all samples were analyzed in triplicates. The limit of detection and the limit of quantification of the analysis was 50 and 125 ng/ml, respectively.

The PK parameters, AUC, Cmax, time to reach Cmax (Tmax), and half-life (*t*_1/2_) were calculated using the R 3.5.1 version (12) and Prism 8.0.1 version (GraphPad Software, Inc., La Jolla, CA, United States) considering a non-compartmental study model.

The AUC and Cmax values were also adjusted for BW and referred to as AUCw and Cmax/w.

## Statistical Analysis

To determine PK parameters, analysis of variance (ANOVA) with Tukey’s or Dunnett’s multiple comparisons test was employed.

To test the correlation between variables, we performed several linear regression analyses. AUC, Cmax, Tmax, and *t*_1/2_ were sequentially used as dependent variables, while age, sex, and alternatively BW or BMI were introduced as independent variables.

All values were expressed as mean and standard deviation (SD). A *p* < 0.05 was considered statistically significant. Statistical analysis was performed using STATA 16 version.

## Results

The LD concentrations were measured in plasma from 35 Caucasian patients (19 men and 16 women) with PD. All of them were LD-naïve patients and received a single dose of oral LD/benserazide (100/25 mg) formulation.

The study population appeared to be homogenous for the age and duration of the disease. No differences were found in BMI median value between men and women, while women had a median value of BW lower than men. Daily energy consumption and lean mass were higher in men than in women, while women showed higher fat mass compared with men without reaching a statistical significance. There was no difference in PD symptoms, monoamine oxidase-B inhibitors (iMAO-B), dopamine agonists (DA) use, and comorbidities between genders. The main characteristics of the study population are listed in Table 1. No differences were found in dietary habits (data not shown).

**TABLE 1:** Clinical characteristics of the study population.

	Men (*n* = 19)	Women (*n* = 16)	*P*-value
Age (years)	61 (±8.7)	62 (±11.8)	NS
BW median value (Kg)	80	70	0.0228
BMI median value (Kg/m^2^)	26	26	NS
Duration of the disease (months)	34 (±28.5)	59 (±24.5)	NS
Daily energy consumption (Kcal)	1798.688 (±381)	1330.875 (±319)	0.0069
Lean mass (Kg)	62.737 (±12.5)	46.7 (±4.3)	0.0021
Fat mass (Kg)	20.125 (±16.3)	25.3 (±9)	NS
**Motor symptoms:**			
Bradykinesia	16 (100%)	14 (100%)	NS
Rigidity	16 (100%)	13 (92,8%)	NS
Tremor	15 (93,75%)	13 (92,8%)	NS
Postural instability	1 (6,25%)	1 (7,14%)	NS
**Antiparkinsonian drugs use:**			
DA	12 (63,1%)	6 (37,5%)	NS
iMAO-B	11 (58%)	6 (37,5%)	NS
DA and iMAO-B	9 (47,3%)	5 (31,2%)	NS
No DA and iMAO-B	5 (26,3%)	9 (56%)	NS
**Comorbidities:**			
Arterial hypertension	7 (43%)	7 (50%)	NS
Hypercholesterolemia	2 (12,5%)	4 (28,6%)	NS
Chronic gastritis	2 (12,5%)	2 (14,2%)	NS
Type II diabetes	2 (12,5%)	0 (0%)	NS

*Except for body weight (BW) and body mass index (BMI), the values are expressed as mean and standard deviation (SD) or number and percentage of patients.*

*Abbreviations: BW, body weight; BMI, body mass index; DA, dopamine agonists; iMAO-B, monoamine oxidase-B inhibitors.*

**FIGURE 1:**
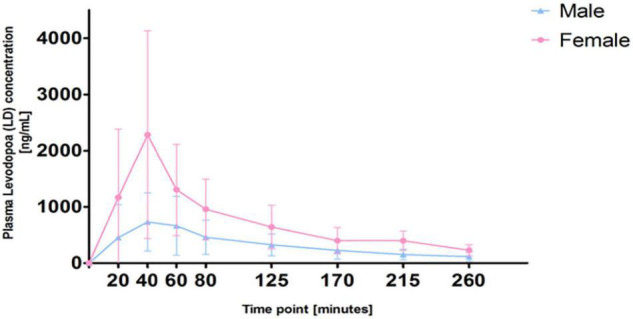
Differences in plasma levodopa (LD) concentrations between men and women.

The differences in plasma LD concentrations between men and women over time are shown in Figure 1. The mean values measured in men and women at each time point (20–260 min) and PK parameters are reported in Table 2.

**TABLE 2:** Plasma levodopa (LD) concentrations and pharmacokinetics (PK) parameters, unadjusted and adjusted by body weight, measured in men and women.

Plasma LD concentration measured at each time point (ng/mL)				Plasma LD concentration measured at each time point corrected by BW (ng/mL)/w			
Time (min.)	M	W	*P*-value	Time (min.)	M	W	*P*-value
20	454.1053 ± 5583.694	1168.42 ± 11220.535	0.0313	20	5.936316 ± 0.93631635	18.14667 ± 819.6273	0.0189
40	732.1368 ± 32.136835	2284.08 ± 1846.953	0.0014	40	9.411579 ± 0.6.893599	34.02933 ± 426.90424	0.0005
60	662.7263 ± 62.726324	1305.037 ± 305.03724	0.0080	60	8.817368 ± 0.8.099045	19.63 ± 912.91515	0.0049
80	459.0368 ± 59.036815	957.55 ± 57.556815	0.0016	80	5.974211 ± 0.4.441285	14.315 ± 48.191977	0.0005
125	324.9421 ± 24.942177	640.9813 ± 4385.7052	0.0035	125	4.402105 ± 0.3.1499	9.516875 ± 0.5.957967	0.0027
170	228.0568 ± 2159.6823	400.2706 ± 00.270623	0.0134	170	3.094211 ± 0.2.54934	5.9375 ± 0.3.454692	0.0085
215	153.3053 ± 53.305392	398.5067 ± 98.506792	0.0000	215	2.066842 ± 0.1.515395	5.848 ± 2.600281	0.0000
260	115.5556 ± 15.555628	229.1933 ± 296.75774	0.0008	260	1.572222 ± 0.1.235321	3.414 ± 0.1.520925	0.0006

**PK parameters**				**PK parameters corrected by BW**			

AUC mcg*h/mL	1.126805 ± 0.126805et	2.583994 ± 0.51.49891	0.0006	AUC/w (mcg*h/mL)/w	0.0147789 ± 0.00996	0.0387062 ± 0.0.02404	0.0385
Cmax ng/mL	909.8947 ± 09.89474 4	2405.094 ± 405.09447	0.0014	Cmax/w (ng/mL)/w	12.15474 ± 2.15474	36.31375 ± 6.31375w	0.0004
Tmax min.	45.26316 ± 5.26316w	46.25 ± 6.25316w	NS				
*t*_1/2_ hours	1.533158 ± 0.0.534977	1.494375 ± 0.1.237298	NS				

*Values are expressed as mean ± SD.*

*Abbreviations: LD, levodopa; BW, body weight; PK, pharmacokinetics; AUC, area under the curve; Cmax, maximum plasma concentration; Tmax, time to reach Cmax; t_1/2,_ half-life; AUCw, AUC adjusted for body weight; Cmax/w, Cmax adjusted for body weight.*

**FIGURE 2:**
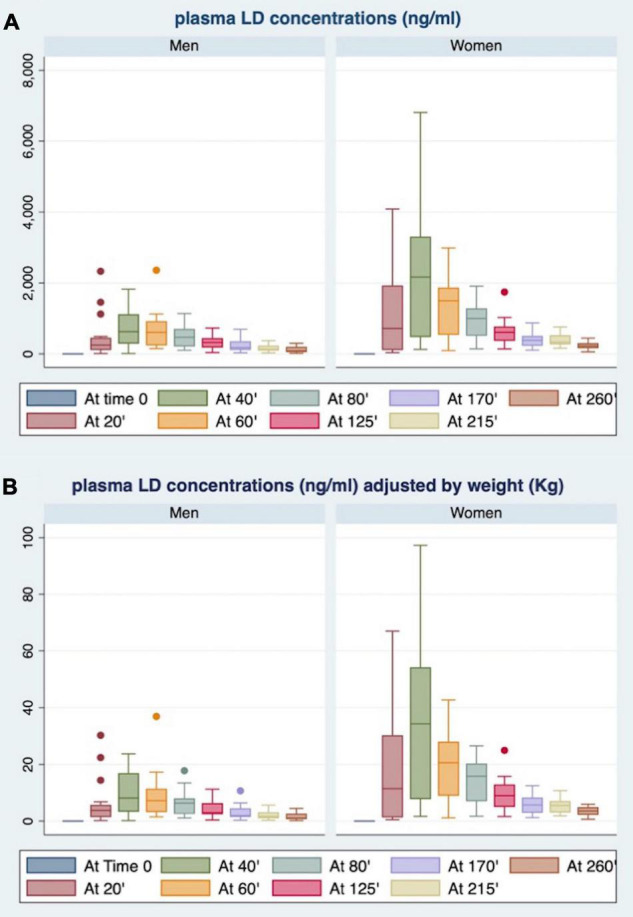
Gender differences in plasma LD concentrations at each time point, unadjusted **(A)** and adjusted for body weight (BW) **(B)**.

As shown in Figure 2, women showed plasma LD concentrations, unadjusted (panel A) and adjusted for BW (panel B), higher than men at each time point.

**FIGURE 3:**
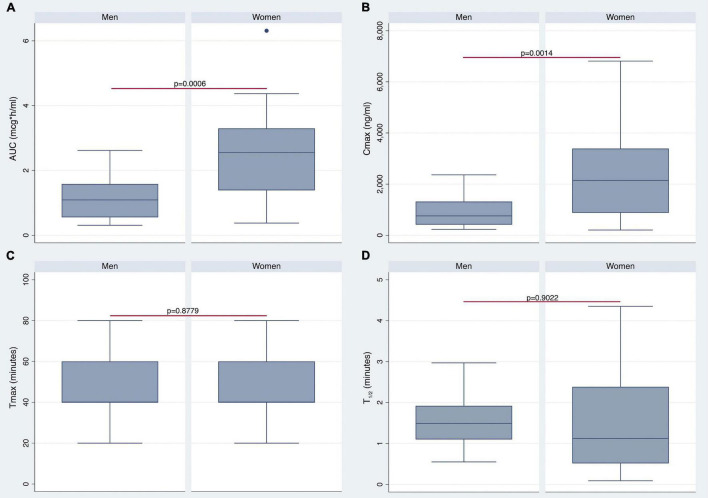
Differences in pharmacokinetics (PK) parameters between men and women. Gender differences in area under the curve (AUC), maximum plasma concentration (Cmax), Tmax, and *t*_1/2_ are shown in panels **(A–D)**, respectively.

**FIGURE 4:**
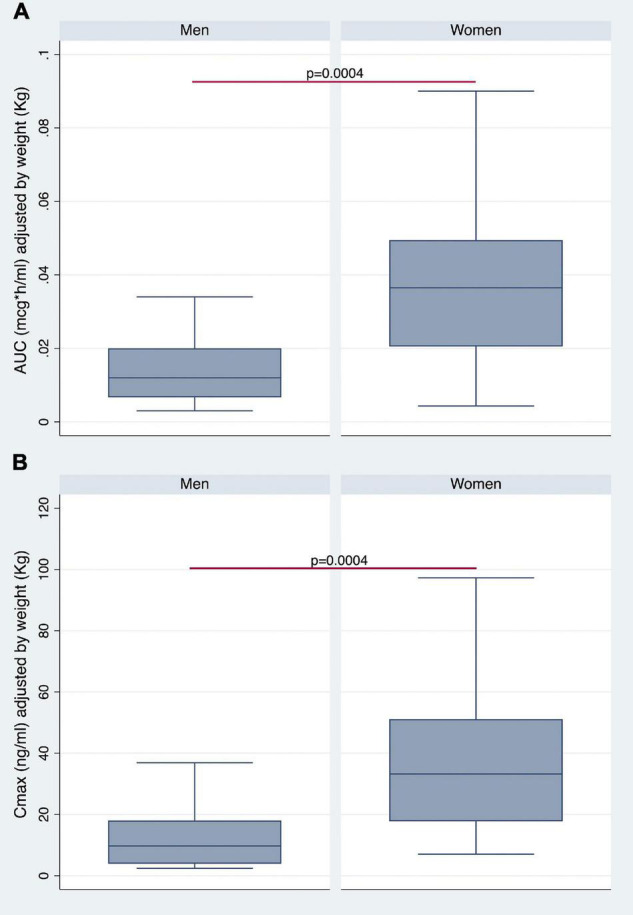
Gender differences in AUC **(A)** and Cmax **(B)** adjusted for BW.

The AUC (Figure 3A) and AUCw (Figure 4A) were higher in women than in men (*p* < 0.0006 and *p* < 0.0004, respectively). As shown in Figures 3B, 4B, women also revealed higher Cmax and Cmax/w when compared with men (*p* < 0.0014 and *p* < 0.0004, respectively). Conversely, there were no statistically significant differences in Tmax and *t*_1/2_ (Figures 3C,D).

**FIGURE 5:**
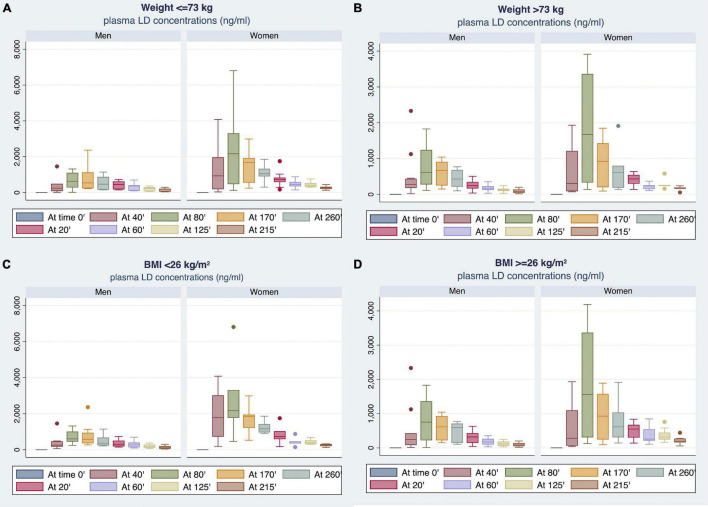
Gender differences observed in plasma LD concentrations by stratifying the study population according to median BW **(A,B)** or body mass index (BMI) **(C,D)** values.

By stratifying the study population according to median BW (i.e., 73 Kg) or BMI (i.e., 26) values, women showed higher plasma LD concentrations compared with men (Figure 5). Notably, BMI median value was 26 for both men and women. The most relevant differences emerged by comparison between men and women with BMI of <26 (Figure 5C).

Table 3 reports the differences in plasma LD concentrations, measured at each time point (20–260 min), and PK parameters between men and women stratified by BMI median value. Women with BMI < 26 showed plasma LD concentrations significantly higher than men with BMI of <26. Considering BMI of ≥26, women demonstrated higher LD concentrations as compared with men in the range of 20–60 min after LD administration without reaching a statistical significance. By contrast, the concentration levels measured in the range of 80–260 min were significantly higher in women as compared with men (Table 3).

**TABLE 3:** Differences in plasma LD concentrations and PK parameters between men and women stratified by the BMI median value.

BMI < 26				BMI ≥ 26			

Plasma LD concentration measured at each time point (ng/ml)							

**Time (min.)**	**M**	**W**	***P*-value**	**Time (min.)**	**M**	**W**	***P-* value**
20	411.1375 ± 11.1375D	1812.371 ± 812.371D	0.0168	20	485.3545 ± 85.3545D	604.962576 ± 04.962576	NS
40	707.75 ± 07.75 576	2785.6 ± 785.6 576	0.0121	40	749.8727 ± 49.872776	1845.25 ± 845.25 76	NS
60	798.425 ± 98.425 76	1732.271 ± 732.27176	0.0262	60	564.0364 ± 64.036476	972.7444 ± 72.744476	NS
80	487.275 ± 87.275476	1227.457 ± 227.45776	0.0010	80	438.5 ± 38.505776	747.6222 ± 47.622276	NS
125	357.7 ± 215.7421	802.7571 ± 02.757121	0.0367	125	301.1182 ± 01.118221	515.1556 ± 15.1556 1	0.0345
170	298.025 ± 98.0256 1	444.2714 ± 44.2714 1	NS	170	177.1709 ± 77.1709 1	366.0478 ± 66.0478	0.0292
215	198.9875 ± 98.9875 1	433.35 ± 33.35 5	0.0056	215	120.0818 ± 68.60688	375.2778 ± 192.1556	0.0006
260	140.05 ± 40.05 155	249.75 ± 80.20506	0.0441	260	95.96 ± 5.961 506	215.4889 ± 15.4889 6	0.0082

**PK parameters**							

AUC mcg*h/mL	1.216038 ± 0.216038me	3.332286 ± 0.332286 et	0.0050	AUC mcg*h/mL	1.061909 ± 0.061909 et	2.001989 ± 0.001989 e	0.0385
Cmax ng/mL	941.5125 ± 41.5125 et	2986.786 ± 986.786	0.0149	Cmax ng/mL	886.9 ± 86.9 6 e	1952.667 ± 952.667 et	0.0474
Tmax min.	57.5 ± 7.574 7 e	40 ± 0 5	NS	Tmax min.	38.18182 ± 8.18182 e	62.22222 ± 0.22222 e	NS
*t*_1/2_ hours	1.4225 ± 0.4225 2 e	1.051429 ± 0.051429 e	NS	*t*_1/2_ hours	1.613636 ± 0.613636 et	1.838889 ± 0.838889 e	NS

*Values are expressed as mean ± SD.*

*Abbreviations: LD, levodopa; PK, pharmacokinetics; AUC, area under the curve; Cmax, maximum plasma concentration; Tmax, time to reach Cmax; t_1/2_, half-life.*

**FIGURE 6:**
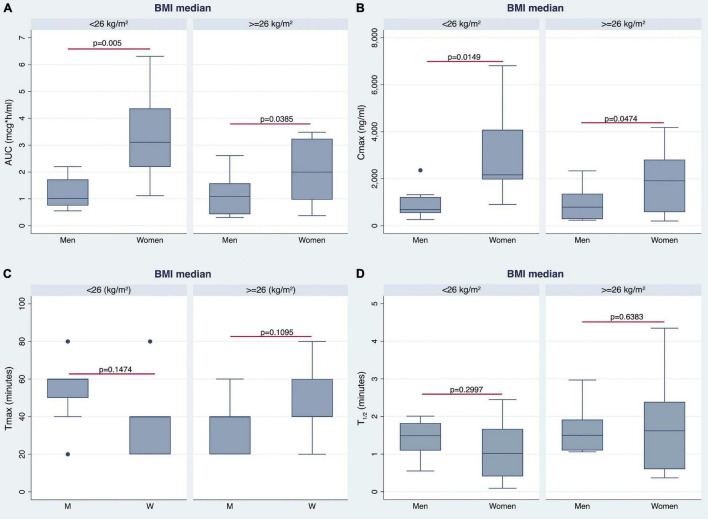
Gender differences observed in AUC **(A)**, Cmax **(B)**, time to reach Cmax (Tmax) **(C)**, and *t*_1/2_ **(D)** by stratifying the study population according to median BMI value.

**FIGURE 7:**
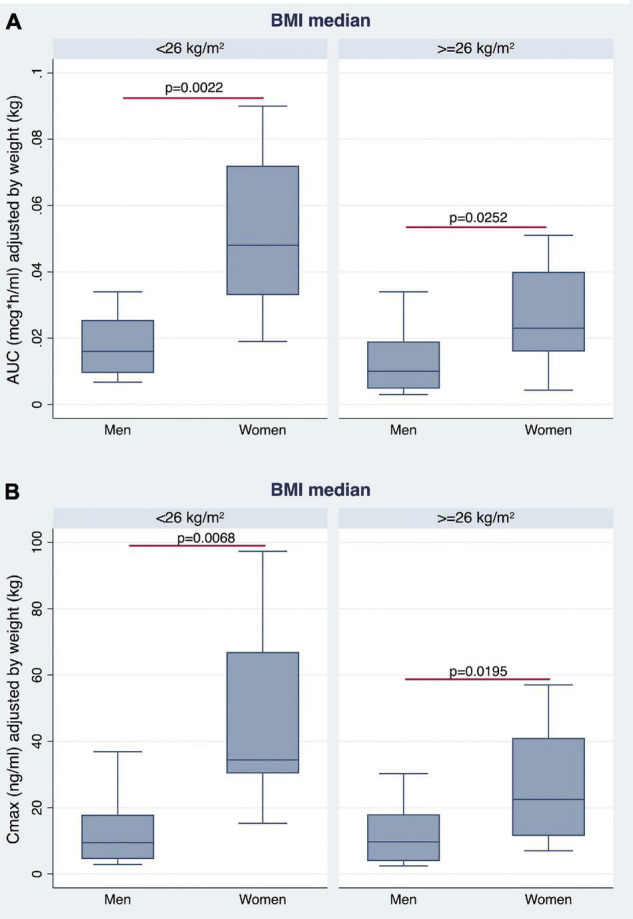
Gender differences observed in AUC **(A)** and Cmax **(B)**, adjusted for BW, by stratifying the study population according to median BMI value.

The AUC and Cmax were significantly higher in women than in men irrespective of BMI stratification (Table 3 and Figures 6A,B). A similar finding was observed considering values adjusted for BW (Figures 7A,B). No gender differences were found regarding Tmax and *t*_1/2_ neither for BMI < 26 nor BMI ≥ 26 values (Table 3 and Figures 6C,D).

### Linear Regression Analyses

Multiple linear regression analyses were sequentially performed to test the predictors of AUC, Cmax, Tmax, and *t*_1/2_ using age, sex, and BW as independent variables. The first analysis tested if age, sex (female), and BW significantly predicted AUC. The fitted regression model was: 4.729196 −0.0220806* (Age) + 1.181306* (female sex) −0.0279986* (BW). The overall regression was statistically significant [*r*2 = 0.3841, *F*(3,31), *p* = 0.0016]. It was found that only the female sex significantly predicted AUC (β = 1.181306, 95% CI 0.3589598 2.003652; *p* = 0.006).

The second analysis tested if age, sex (female) and BW significantly predicted Cmax. The fitted regression model was: 3,889.778 −23.72423* (Age) + 1,314.009* (female sex) −19.01502* (BW). The overall regression was statistically significant [*r*2 = 0.3131, *F*(3,31), *p* = 0.0080]. It was found that only female sex significantly predicted Cmax (β = 1,314.009, 95% CI 367.4519 2,260.566; *p* = 0.008).

The third linear regression analysis, testing if age, sex (female), and BW significantly predicted Tmax, did not identify any significant association. The fitted regression model was: 37.0304 + 0.2527491* (Age) −0.124896* (female sex) −0.0896137* (BW). The overall regression was not statistically significant [*r*2 = 0.0274, *F*(3,31), *p* = 0.8318].

The same results were found by considering *t*_1/2_. The fitted regression model was: −1.265975 + 0.0264122* (Age) + 0.0958726* (female sex) + 0.0147279* (BW). The overall regression was not statistically significant [*r*2 = 0.1020, *F*(3,31), *p* = 0.1020].

**FIGURE 8:**
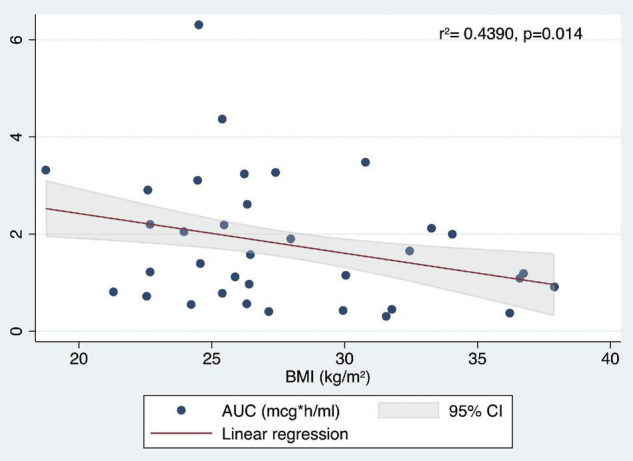
Correlation between AUC (mcg*h/ml) values and BMI (kg/m^2^).

Another set of multiple linear regression analyses was performed to test the predictors of sequentially AUC, Cmax, Tmax, and *t*_1/2_ using age, sex, and BMI (instead of BW) as independent variables. The first analysis tested if age, sex (female), and BMI significantly predicted AUC. The fitted regression model was: 4.531093 −0.0123913* (Age) + 1.559116* (female sex) −0.0970631* (BMI). The overall regression was statistically significant [*r*2 = 0.4390, *F*(3,31), *p* = 0.0004]. It was found that female sex (β = 1.559116, 95% CI 0.8314479 2.286785; *p* < 0.0001) and BMI (β = −0.0970631, 95% CI −0.1733004 −0.0208258; *p* = 0.014) significantly predicted AUC (Figure 8).

The second analysis tested if age, sex (female), and BMI significantly predicted Cmax. The fitted regression model was: 4,083.443 −16.89948* (Age) + 1,582.499* (female sex) −78.50151* (BMI). The overall regression was statistically significant [*r*2 = 0.3542, *F*(3,31), *p* = 0.0032]. It was found that only female sex significantly predicted Cmax (β = 1,582.499, 95% CI 731.581 2,433.417; *p* = 0.001).

The third linear regression analysis, testing if age, sex (female), and BMI significantly predicted Tmax, did not identify any significant association. The fitted regression model was: 19.30049 + 0.2710341* (Age) + 0.4642899* (female sex) + 0.3447133* (BMI). The overall regression was not statistically significant [*r*2 = 0.0318, *F*(3,31), *p* = 0.7972].

**FIGURE 9:**
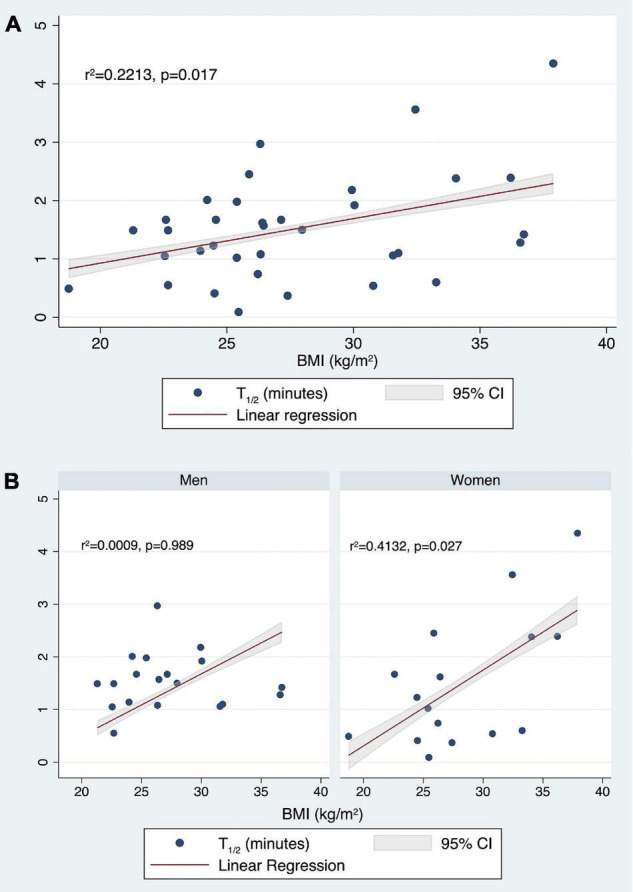
Correlation between *t*_1/2_ (min) values and BMI (kg/m^2^) in the whole population **(A)** and stratifying by sex **(B)**.

Finally, considering *t*_1/2_, the fitted regression model was −1.802667 + 0.0208382* (Age) −0.1261094* (female sex) + 0.0756267* (BMI). The overall regression was statistically significant [*r*2 = 0.2213, *F*(3,31), *p* = 0.0487]. It was found that only BMI significantly predicted *t*_1/2_ (β = 0.0756267, 95% CI 0.0143407 0.1369126; *p* = 0.017) (Figure 9A).

Then, stratifying by gender, we tested if age and BMI predicted the *t*_1/2_. For men, the fitted regression model was 1.660209 −0.0018911* (Age) −0.0004214* (BMI). The overall regression was not statistically significant [*r*2 = 0.0009, *F*(2,16), *p* = 0.9928].

For women, the fitted regression model was −3.432078 + 0.0203022* (Age) + 0.1300486* (BMI). The overall regression was statistically significant [*r*2 = 0.4132, *F*(2,13), *p* = 0.0313]. It was confirmed that in women BMI significantly predicted *t*_1/2_ (β = 0.1300486, 95% CI 0.0172322 0.242865; *p* = 0.027) (Figure 9B).

## Discussion

Despite LD being considered the most effective antiparkinsonian drug since the 1970s, the balance between drug effectiveness and side effects has not yet been determined (1). This is crucial in women who are particularly prone to develop LD-related complications, mainly DYS (7). However, the few available studies focused on the gender-related differences in LD pharmacodynamics, and PK enrolled patients previously treated with LD and with different formulations and dosages (13, 14).

In the present study, plasma concentrations and PK parameters were measured in LD-naïve patients, who received the same LD formulation, and the results were compared between men and women.

Women showed higher levels of AUC and Cmax when compared with men, confirming the few data available in the literature. Notably, plasma LD concentrations were significantly higher in women than in men at each time point, covering a time of 4 h and 30 min.

Gender differences in PK and pharmacodynamics have been investigated in recent times. Oral bioavailability and distribution were reported to exert the most important influence on the PK of several compounds. Such differences seem to be mainly related to BW; however, they can persist also after adjusting for this biometric variable (15).

The data on the differences in LD PK between men and women are scarce and mainly regarding the LD administration routes other than the oral one (14).

Several studies suggested considering BW and BW loss during therapy because of an inverse relationship between plasma LD levels and BW (16, 17). Martinez-Ramirez et al. (18) described women in a subgroup of extremely sensitive patients reporting a brittle response, defined as the presence of highly disabling DYS after LD standard doses. No less important is the fact that these women have lower BW than patients who were better responders (18).

Conversely, Kumagai et al. (9), in a population of elderly Japanese patients on LD chronic treatment, reported that women had a significantly greater LD bioavailability compared with men irrespective of BW. This finding is consistent with our results, obtained in LD-naïve patients after the first LD administration, showing that plasma concentrations, as well as AUC and Cmax, were higher in women than in men, also after adjusting for BW. We found that AUC and AUCw were 2.29 times and 2.62 times higher in women than in men, respectively. Similar behavior was found for Cmax and Cmax/w, which were 2.64 and 2.98 times higher in women than in men, respectively.

Multiple linear regression analyses showed that the female sex was the only predictor of AUC and Cmax. No correlation was found neither with Tmax nor with *t*_1/2_.

Stratifying the study population according to the BMI median value (i.e., 26 for both women and men), it was possible to highlight the most important differences between lighter women and lighter men, especially in the time range of 20–80 min corresponding to LD peak. Women demonstrated higher values than men also in the group with a BMI of ≥26. However, significant differences were found only in the time range of 125–260 min, corresponding to through PK.

It is of particular interest that, using BMI as an independent variable instead of BW, a significant association was found with *t*_1/2_, as well as with AUC and Cmax. The best predictors of AUC were female sex (*p* < 0.0001) and BMI (*p* = 0.014), Cmax was predicted only by the female sex, while only BMI significantly predicted *t*_1/2_. Higher BMI was associated to lower AUC and higher *t*_1/2_ values. Moreover, stratifying by sex, BMI was confirmed to significantly predict *t*_1/2_ in women, but not in men.

This is an important finding, especially considering that, after repeated drug intake, both wearing-off and DYS are associated with the short plasma half-life of LD. As a matter of fact, stabilizing the plasma LD levels is considered the best way to attenuate these adverse events (1).

Besides the low BW values of female patients, other variables have been considered to explain gender differences in the LD PK and LD effects.

Women show a slower gastric emptying time compared with men (19) and the variability of transit times is crucial for drugs with pronounced intestinal regional differences in absorption, such as LD (20). Moreover, it has been reported that women have about 25% lower catechol-*O*-methyltransferase (COMT) enzyme activity than men (21).

Some studies evaluating the possible influence on LD PK of other antiparkinsonian drugs failed to demonstrate pharmacokinetic-drug interactions (8). In the present study, there was no statistically significant difference in the use of DA and/or iMAO-B between women and men. However, given the small sample size, further studies are required to confirm this finding. Nonetheless, this is the first study assessing gender differences in LD PK in LD-naïve patients with PD and suggests that gender significantly affects LD PK parameters since the first intake. Another strength of the study is the prospective design and the homogeneity of the study population regarding age, disease duration, and use of other antiparkinsonian drugs.

Adjusting plasma LD levels by BMI allowed us to observe the most relevant pharmacokinetic differences according to gender. This biometric variable, even more than the BW, could be useful to set up the best-personalized approach. In particular, BMI emerged as the only predictor of *t*_1/2_ in women and not in men. Thus, it is important considering that this finding regards patients receiving LD for the first time.

## Conclusion

Taken together, our findings provide novel insights into gender differences in LD pharmacokinetics, possibly contributing to the later development of motor complications and dyskinesia in PD. The results refer to parameters measured at the first drug intake of patients enrolled in an ongoing study with a 2-year follow-up. Future analyses will allow us to assess whether the highlighted differences translate into different patterns of adverse events in men and women.

## Data Availability Statement

The raw data supporting the conclusions of this article will be made available by the authors, without undue reservation.

## Ethics Statement

The studies involving human participants were reviewed and approved by Comitato Etico Campania Sud-A.S.L. Napoli 3. The patients/participants provided their written informed consent to participate in this study.

## Author Contributions

VC conceptualized the study and drafted the manuscript. VI conceptualized the study and oversaw the data analysis. MR, MP, MA, CS, AN, IC, and CC enrolled the patients. ED participated in data analysis and drafting of the manuscript. BC and VG performed the experiments and data analysis. GS performed the experiments. GC performed data analysis and participated in the drafting of the manuscript. PB and AF oversaw the drafting of the manuscript. MTP conceptualized the study and oversaw the drafting of the manuscript. All authors contributed to the article and approved the submitted version.

## Conflict of Interest

The authors declare that the research was conducted in the absence of any commercial or financial relationships that could be construed as a potential conflict of interest.

## Publisher’s Note

All claims expressed in this article are solely those of the authors and do not necessarily represent those of their affiliated organizations, or those of the publisher, the editors and the reviewers. Any product that may be evaluated in this article, or claim that may be made by its manufacturer, is not guaranteed or endorsed by the publisher.

## Abbreviations

LD: levodopa
DDCI: dopa-decarboxylase inhibitors
PD: Parkinson’s disease
MNMF: motor/non-motor fluctuations
DYS: dyskinesias
PK: pharmacokinetics
BW: body weight
AUC: area under the curve
Cmax: maximum plasma concentration
MDS: Movement Disorder Society
EDTA-2Na: ethylenediaminetetracetic acid disodium
UHPLC-MS: ultra-high performance liquid chromatography coupled with mass
TFA: trifluoroacetic acid
HFBA: heptafluorobutyric acid
ISTD: internal standard
PFP: pentafluorophenyl
Tmax: time to reach Cmax
*t*_1/2_: half-life
AUCw: AUC adjusted for body weight
Cmax/w: Cmax adjusted for body weight
BMI: body mass index
iMAO-B: monoamine oxidase-B inhibitors
DA: dopamine agonists
COMT: catechol-*O*-methyltransferase.
